# The Academic Pill: How Academia Contributes to Curing Diseases

**DOI:** 10.1177/2472555218824280

**Published:** 2019-02-20

**Authors:** Marc Bickle

**Affiliations:** 1 Technology Development Studio, Max Planck Institute of Molecular Cell Biology and Genetics, Dresden, Germany

This special issue of *SLAS Discovery* showcases academic screening centers and not-for-profit translational drug discovery centers. Historically, high-throughput screening was developed by the pharmaceutical industry and until the end of the 20th century was mainly carried out within its walls. After the sequencing of the human genome, academic institutions started creating screening centers to either find tool compounds or carry out RNA interference screens to study the genome. In 2003, the U.S. National Institutes of Health (NIH) Roadmap set out a plan for creating screening centers and chemical libraries, further strengthening the trend of academic screening.^1^ Although many academic screening centers aim to discover tool compounds for chemical biology, an appetite has always existed for more translational projects; academic screening would hopefully produce therapeutic molecules that could be used in the clinic. The desire for novel therapeutic molecules was particularly strong for neglected and rare diseases, where a lack of economic incentive meant that therapies were not available. Like many trends, after an initial enthusiasm there came a realization that developing new drugs was challenging and that academic screening centers, while facing different difficulties compared with industrial screening laboratories, also struggled to bring drugs into the clinic.

The reasons for this struggle are numerous, but I would like to underline two major reasons. First, there is a lack of funding opportunities for bringing hit compounds forward and developing lead candidates into drug candidates. Public research funds typically do not finance such work, since it is not basic research, and the projects are at too early a stage for applying for translational grants to spin out a company. It is equally hard to find pharmaceutical partners at this stage. Typically, the pharmaceutical industry invests in projects where a proof of principle has been obtained in animals, and the effects of the molecule of interest are better understood. Second, academic screening centers typically lack the infrastructure to host, curate, and process very large, high-quality chemical collections. Given the importance of the quality of compounds entering the screening process, it is perhaps not surprising that many projects did not materialize into lead molecules that could be brought forward into the clinic.

To fill the gap between academic research and drug development, translational drug discovery centers were established. These centers were created to help bridge academia and industry; to have the critical mass in terms of people, instruments, compounds, and chemistry; and to form private–public partnerships (PPPs) to bring innovative compounds forward into the clinic.

This special issue of *SLAS Discovery* offers a snapshot of the research being conducted in academic screening laboratories and in translational drug discovery centers. We invited many centers to contribute and 15 laboratories answered the call. As to be expected, the 15 manuscripts submitted cover a wide range of topics. The first three manuscripts, by Franke et al.,^2^ Warchal et al.,^3^ and Janosch et al.,^4^ describe analytical methods for phenotypic profiling of cellular responses. Using phenotypic signatures for discovering the mode of action of compounds is a very active research field, and it is not surprising to see that all three analytical articles focus on that subject. The manuscripts of Starkuviene et al.,^5^ Imamura et al.,^6^ Colussi et al.,^7^ Close et al.,^8^ Siva et al.,^9^ and Wiseman et al.^10^ all describe novel model systems, assays, and technologies that allow the screening of large collections of molecules. This illustrates that academia is a rich source for novel assays as basic research is transformed into screens, leading to unexplored therapeutic avenues. Lastly, Baillargeon et al.,^11^ Moraes et al.,^12^ Otvos et al.,^13^ D’Agostino et al.,^14^ Birchall et al.,^15^ and Brennecke et al.^16^ are reviews of the screening efforts of screening laboratories and their networks, covering a wide range of applications. These reviews showcase how expertise in different fields allows academic projects to progress toward the clinic.

## Contributing Laboratories

The 15 laboratories that contributed to this special issue all have individual expertise and operational modes, as summarized below.

The **Helmholtz Centre for Infection Research** (HZI) in Braunschweig, Germany, started its operations in 2006, although its predecessor institute was founded in 1965.^2^ The institute currently has 822 employees, and the three main goals of the Chemical Biology (CBIO) Department are discovering new antibacterial and antiviral drugs, characterizing their functionality, and optimizing their properties. CBIO focuses on infection research and small molecules that can function as antimicrobial or antiviral agents, interfere with pathogenicity factors, or stimulate the immune system. The discovery of new drugs includes the development of innovative, mainly phenotypic screening assays for medium-throughput screening campaigns. At the department’s disposal are approximately 30,000 compounds, of which the proprietary HZI natural product collection and a proprietary academic collection (approximately 4000 compounds) are specific features. The department is actively involved in several projects of the German Centre for Infection Research (DZIF), in the Translational Unit “Antibiotics” and the Translational Infrastructure “Antivirals.” Screening is conducted either at the HZI or at an external partner site. A medium- to high-throughput screen under biological safety level S3 or S1 conditions can be performed with a robotic pipetting system. Antibacterial or antiviral screens under S2 conditions will become operative in H1/2019. Identified active compounds are profiled against the ESKAPE panel of bacterial pathogens and against mammalian cell lines to determine the selectivity index. For mode of action studies, various functional and profiling methods are established to characterize the effect of the compounds on the target pathogen or cell line. These include membrane potential and membrane permeability, high-content imaging, impedance spectroscopy,^2^ transcriptomics, targeted and untargeted metabolomics, and peptide arrays as the main “omics” technologies. Specific technologies for studying the uptake of compounds in gram-negative bacteria have been established. The department also has synthetic chemistry capabilities (approximately 12 FTE) that deal with lead generation and lead optimization by synthesis. In addition to de novo-designed drug conjugates and natural product-based lead optimization, the team advances screening actives to hit series. The most promising compounds are profiled in vivo in the animal facility of the HZI, where mouse models to study the pharmacokinetic (PK) and pharmacodynamic (PD) parameters of advanced compounds in antibacterial or antiviral infection models have been set up.

The laboratory is run under an open-access model with internal and external users under a research collaboration contract.

Website: https://www.helmholtz-hzi.de/broenstrup

Email: mark.broenstrup@helmholtz-hzi.de

The **Edinburgh Cancer Discovery Unit** (ECDU) is an academic research group located within the Cancer Research UK Edinburgh Centre, MRC Institute of Genetics and Molecular Medicine, University of Edinburgh in Scotland, United Kingdom.^3^ The ECDU was founded in 2011 as a not-for-profit activity to provide a multidisciplinary group of core skills embracing advanced technology platforms and disease models, which drive innovations in oncology drug discovery and development. The ECDU research staff currently comprises Professor Neil Carragher (director), seven senior scientists, and three full-time postgraduate PhD students. Key technologies used within the unit include high-content imaging, confocal and multiphoton confocal imaging, image analysis, reverse-phase protein array, and NanoString (Seattle, WA) molecular profiling of transcriptomic and posttranslation pathway networks. The ECDU mission is to develop and apply novel genetically defined 2D and 3D cell- and tissue-based assays that represent advances over the current state of the art in disease relevance and inform subsequent preclinical and clinical development strategies. The research unit is highly proficient in image-based phenotypic screening using predominantly small-molecule chemical libraries (e.g., approved drugs, annotated chemogenomic probe compounds, and diverse lead-like chemical libraries) and, through partnerships, therapeutic antibodies and peptides. The laboratories are equipped with the latest kinetic (IncuCyte Zoom, Essen BioScience, Sartorius, Göttingen, Germany) and high-content (ImageXpress-microXL, Molecular Devices, LLC, Sunnyvale, CA) screening platforms, fully integrated with plate handling robotics, barcode sample tracking, and bespoke multiparametric image analysis/informatics workflows. The unit also routinely employs both forward-phase and reverse-phase protein microarray platforms (Aushon 2470, GeSim Nanoplotter 2.1E, Radeberg, Germany; Innopsys, Carbonne, France; InnoScan 710 IR and Zeptosens; and the NanoString, Seattle, WA n-counter platform) to profile preclinical and clinical samples and drug mechanism of action at transcriptomic and posttranslational pathway network levels. The ECDU works in close collaboration with several pharmaceutical and biotechnology industry partners and academic research groups to identify hit molecules, advance small-molecule lead generation, and classify compound mechanism of action through multiparametric high-content and pathway profiling. The ECDU provides an open-access model to both internal University of Edinburgh research groups and external academic or industry organizations through either fee-for-service or joint research collaboration agreements. The ECDU operates a full-cost recovery model for projects with external partners and recovery of only consumables for Cancer Research UK-funded groups within the University of Edinburgh. Intellectual property (IP) policy is flexible and dictated on a case-by-case basis dependent upon the nature of the project and research agreement (i.e., distinct IP arrangements are considered for fee-for-service or research collaboration agreements). IP arrangements are pre-agreed and documented in the service or research collaboration agreements prior to commencing work with external partners. All contracts and research agreements are arranged through the business development function at Edinburgh Innovations.

Website: https://www.ed.ac.uk/cancer-centre/impact-and-innovation/translational-science/edinburgh-cancer-discovery-unit-ecdu

Email: edinburgh.innovations@ed.ac.uk

The **Technology Development Studio** (TDS) is an academic screening facility that was created in 2004 at the Max Planck Institute of Molecular Cell Biology and Genetics (MPI-CBG) in Dresden, Germany.^4^ The mission of the facility is to provide state-of-the-art cellular screening services for its users and to develop novel technologies where required (hence the name of the facility). The facility specializes in high-throughput microscopy since this technology provides great flexibility and allows in-depth analysis of biological systems. Homogeneous assays such as luminescence or fluorometric measurements and biochemical assays are also run when required. Both genomic and chemical screens can be run, and the facility has several genome-wide RNAi libraries and approximately 130,000 compounds. The TDS has screened a wide diversity of cellular systems ranging from simple immortalized cell lines to primary cells and stem cells both in 2D and 3D. Additionally, the facility has also screened small model organisms such as *Caenorhabditis elegans* and zebrafish. Protocols have been developed for handling 3D nonadherent objects with standard liquid handlers. Furthermore, optical clearing protocols and methods for imaging 3D objects in 384-well plates have been optimized. Like all facilities at the MPI-CBG, the TDS offers its excess capacity to outside users and has carried out screens for many academic and industrial collaborators not associated with the MPI-CBG. Distribution of intellectual property is decided on case-by-case by the users based on the contribution of each of the parties involved. To provide screening services to outside clients, a full-cost accounting system is used that considers salaries, consumables, instrument time, depreciation, and overheads. The TDS helps prepare grants and participates in funding calls to cover the cost of screening. Services range from designing a screening assay from scratch to executing an already optimized assay and running the analysis of the data on the institute’s computer cluster. The data are owned by the client and transferred at the end of the project. To keep the costs minimal, the TDS uses open-source software for its work. Image analysis is mainly carried out with CellProfiler, Fiji, and sometimes bespoke Python image analysis scripts developed for challenging applications. For data mining, the TDS has developed a suite of software tools in the KNIME software platform. KNIME allows building analytical pipelines in a user-friendly graphical interface that helps to visualize the flow of data. The TDS has developed many tools specific for screening applications, such as plate viewers, normalization nodes, data annotations, and population analysis nodes, which can be downloaded from the KNIME website (https://www.knime.com/downloads/download-knime). One very powerful tool that the TDS introduced to the KNIME platform is scripting nodes for R and Python. These allow users to write their code in those programming languages and, with the insertion of a few lines into the code, to generate a graphical user interface in KNIME using RGG (R GUI Generator). In this manner, a computer scientist can rapidly deploy code to scientists who are not comfortable with programming scripts.

Website: http://www.mpi-cbg.de/facilities/profiles/ht-tds.html

Email: bickle@mpi-cbg.de

The **Pharmacy Chemical Biology Center** (PCBC) in the University of Pittsburgh (Pitt) School of Pharmacy was founded in 2011, evolving out of a high-throughput screening (HTS) center that was founded in 2005 with resources from the Schools of Medicine, Pharmacy, and Arts and Science.^8^ The Pittsburgh Molecular Library Screening Center (2005–2008) and the Pittsburgh Specialized Application Center (2009–2012) were partly supported by funding from the National Institute of Health’s (NIH) Roadmap Initiative Molecular Library Pilot Screening Center Network and the National Cancer Institute’s (NCI) Chemical Biology Consortium, respectively. The PCBC was created as part of the School of Pharmacy’s D^4^ initiative to provide one-stop access to drug discovery, development, and delivery expertise in an interactive and collaborative environment. Research faculty in the D^4^ team have a proven track record in drug discovery and preclinical drug development in both the pharmaceutical and academic sectors. The faculty’s complementary and overlapping capabilities encompass the drug discovery and development process and address the major causes of drug failure. Dr. Paul A. Johnston is the principal investigator (PI) of the PCBC and has 28 years of drug discovery experience in the pharmaceutical, biotechnology, and academic sectors. The PCBC is staffed by three full-time research scientists and varying numbers of graduate students or visiting scientists, most recently two PhD students, one MS student, and one visiting postdoc. The Molecular Devices SpectraMax M5e and Envision (PerkinElmer, Waltham, MA) microtiter plate reader platforms provide multimode assay detection capabilities for UV/Vis absorbance, fluorescence intensity, fluorescence polarization, time-resolved fluorescence resonance energy transfer (TR-FRET), homogenous time-resolved fluorescence (HTRF), and luminescence. The Molecular Devices (Sunnyvale, CA) ImageXpress Micro field-based automated high-content screening (HCS) imaging platform, MetaXpress Imaging and Analysis software, and MDCStore database allow for the capture, analysis, and storage of images acquired in transmitted light and/or five fluorescent channels. The PCBC uses the ScreenAble laboratory information management system software to process and analyze compound information and HTS/HCS data. The PCBC has a 10,000-compound nonpeptide peptido-mimetic diversity subset of a 142,000 protein–protein interaction library, and a 50,000-compound diversity library selected from a 410,000 core library, which enables it to effectively sample a compound diversity of 635,500 compounds. The PCBC provides guidance in assay format selection and assistance with assay development, optimization, and implementation of target-based biochemical and cell-based HTS assays, HCS imaging assays, and phenotypic drug discovery screens. The PCBC performs active confirmation, counterscreens in orthogonal assay formats, hit characterization in secondary and tertiary assays, mechanism of action studies, and bioassay support for medicinal chemistry lead optimization efforts. Projects can be loaded into the PCBC portfolio at any of these stages and are supported through a variety of funding sources, including NIH grants awarded to the PI or his collaborators at Pitt or other academic institutions (national and international), donations from philanthropic foundations, and research contracts with other academic institutions and biotechnology or pharmaceutical companies. Project budgets include personnel salaries and benefits, reagents, consumables, and Pitt institutional indirect costs. Depending upon the funding source, any unassigned intellectual property rights are subject to negotiation with the Pitt office of research.

Website: http://www.pharmacy.pitt.edu/directory/profile.php?profile=856

Email: paj18@pitt.edu

The **Stem Cell Hotel** is an innovative collaborative phenotyping unit located within the Centre for Stem Cells and Regenerative Medicine (CSCRM) at King’s College London, United Kingdom.^10^ It is based at the 28th Floor of Guy’s Tower, Great Maze Pond, with spectacular views over the Thames and the city. The center was inaugurated in December 2015 and the Stem Cell Hotel started its operation. A dedicated team of stem cell scientists, imaging experts, and analysts, with help from interns, bioinformaticians, and business advisors, is forming around the project, spearheaded by Dr. Davide Danovi. Technologies from several providers enable microscopy and high-content analysis, cell-based assays, and data integration. The Stem Cell Hotel offers access to high-content imaging (PerkinElmer Operetta and Operetta CLS, PerkinElmer, Waltham, MA; NanoEntek, Julistage, Guro-gu, Seoul, Korea; and Essen BioScience IncuCyte Zoom, Sartorius, Gottingen, Germany) and quantitative phase imaging (Livecyte, Phasefocus, Sheffield, UK) devices. Resources and expertise in the areas of stem cell biology, artificial microenvironments for cell culture, and high-content imaging are provided as services. This includes assistance for assay development, image acquisition, and dedicated data analysis and integration. The Stem Cell Hotel develops robust methods for profiling and benchmarking cells for cell therapy and drug discovery applications. It uses dynamic and endpoint imaging and high-content analysis integrated with genomics and other biological datasets. The operation also leverages expertise from a critical mass of scientists and innovative research projects currently ongoing at the center, such as the development of standard methods for characterization of induced pluripotent stem cells (iPSCs). Created within the framework of the Human Induced Pluripotent Stem Cell Initiative (HIPSCI) and serving the UK Regenerative Medicine Platform (UKRMP), the facility offers external users services ranging from initial training on the instruments to more in-depth assistance in assay development, acquisition, and further data analysis. Its cost model varies from pure charging for the time of use of instruments and consumables to scientific collaborations, from co-development of software and applications with technology providers to contract research-type projects. Importantly, the facility works effectively with technology providers embedding instruments and technologies in the space. This has taken the form of leases, extended demos, beta testing of software, and agreements to offer the possibility to showcase devices to future potential customers. These innovative options foster a constructive dialogue and offer a testing bed for research and industry to understand needs and mature products and solutions. As an example, one of the center’s technology providers can establish a strategic partnership to provide in-house technical support with the use of the entire set of instruments, ranging from assay development to image analysis. The Stem Cell Hotel’s policy sees intellectual property (IP) staying with the user unless otherwise discussed on specific projects that require significant input from the Stem Cell Hotel. Born from research, boldly translational, and embracing the spirit of open innovation, the SCH grants access to state-of-the-art technology and serves communities centered around academic, clinical, and commercial research in a highly collaborative environment.

Website: http://www.kclstemcellhotel.org

Email: davide.danovi@kcl.ac.uk

The **CellNetworks Advanced Biological Screening Core Facility** was established in 2007 as one of the first CellNetworks Core Facilities and is located at Heidelberg University in the BioQuant Center for “Quantitative Analysis of Molecular and Cellular Biosystems” in Heidelberg, Germany.^5^ The facility is equipped with state-of-the art instruments, and its experts possess long-term experience in the field of biologicals, RNAi screening, automated screening microscopy, and data analysis, and lately, CRISPR-mediated gene editing. The facility offers support in assay development, automated sample preparation, and high-throughput solid-phase-based transfection in multiwell plates or cell microarrays. A number of focused and genome-wide libraries of siRNAs, microRNAs, cDNAs, and crRNAs are available. In addition, the facility facilitates the contact between customers and experts on the campus to help establish collaborations and strengthen the research network in the field of biological screening in Heidelberg. In this manner, pilot projects starting out as services within the facility are developed into successful multilateral collaborations. The facility has either been a partner with or played a leading role in several projects funded by the European Union, German Federal Ministry of Education and Research (BMBF), and Baden Württemberg Stiftung. Recently, the High-Content Analysis of the Cell (HiCell) group was established with the aim to design, develop, and apply novel technologies for high-content screening and analysis. Once tested and standardized, the novel technologies are incorporated into the portfolio of the Advanced Biological Screening Facility and become accessible for users. HiCell focuses on the development of methodologies to interfere with cell function on DNA, mRNA, and protein levels, on the miniaturization of cellular assays such as the cell microarrays presented in this issue,^5^ and on combinatorial assays. The facility also offers correlative microscopy combining high-speed and super-resolution imaging as well as 3D assays and imaging. Lately, it has been focusing on single-cell analysis.

Websites:

Advanced Biological Screening Facility: http://www.bioquant.uni-heidelberg.de/index.php?id=42

High-Content Analysis of the Cell (HiCell): http://www.bioquant.uni-heidelberg.de/research/groups/high-content-analysis-of-the-cell-hicell.html

Email: holger.erfle@bioquant.uni-heidelberg.de

The **Drug Discovery Initiative** at the University of Tokyo, Japan, aims to promote academic research and innovation in drug discovery. It supports academic and industrial researchers who want to screen chemical samples to find either chemical biological tools, drug leads, or agrochemicals.^6^ The Drug Discovery Initiative has constructed a chemical library consisting of about 280,000 samples chosen primarily on druggability and structural diversity. The collection includes 63,000 samples deposited by industry since 2006. These chemicals are provided (in assay-ready plates if required) to researchers in Japan who are willing to disclose their research goals and report their assay results to the initiative under a confidentiality agreement. The users are required to pay the consumables and shipping costs, while the chemical samples themselves are free of charge. The initiative does not claim any rights to the results of the screens in the absence of intellectual contribution. The initiative has provided more than 22 million samples to more than 500 users so far. The initiative has various screening instruments that are available to users. Consultation and technical assistance can also be provided as the majority of the 25 employees have research experience in pharmaceutical companies. Short training courses on chemical screening are held several times a year to help beginners. More professional tutorials focusing on specific themes are also held in cooperation with the Conference on Biomolecular Screenology, where everybody, from beginner to expert, is welcome to attend. Recently our organization founded the Lead Exploration Unit, which has started a hit-to-lead synthesis service, as well as ADMET support, upon request.

Website: https://www.ddi.u-tokyo.ac.jp/en/

Email: ddiinfo@mol.f.u-tokyo.ac.jp

The **Moulder Center for Drug Discovery Research** is located at the Temple University School of Pharmacy in Philadelphia, Pennsylvania.^7^ The center was established in 2008 thanks to a generous gift from School of Pharmacy alumni Lonnie and Sharon Moulder. The mission of the Moulder Center is focused on the discovery of new clinical candidates. Furthermore, the center provides education and training for students, postdoctoral fellows, and faculty in the application of modern drug discovery techniques. The center participates in collaborative research within Temple University as well as externally with research institutions, universities, and the pharmaceutical industry. The Moulder Center embraces three business models: collaborative research, contract research, and consulting/providing expertise and oversight to external resources. The Moulder Center is a fully integrated academic drug discovery laboratory with resources and capabilities in assay development and high-throughput screening, in vitro pharmacology medicinal chemistry, in vitro ADME, and in vivo pharmacokinetics. The center is staffed with faculty, research associates, postdoctoral fellows, and graduate students. Most Moulder Center members are highly skilled in drug discovery and have accrued many years of experience working in the pharmaceutical industry. High-throughput screening is focused on small-molecule collections with automation supported by Janus workstations (PerkinElmer, Waltham, MA). Compound and data management utilizes the Dotmatics informatics suite (Bishop’s Stortford, Herts, UK). The center maintains an ADME screening panel of in vitro assays to assess drug-like properties of candidate compounds. These assays measure hERG inhibition, aqueous solubility, chemical stability, stability in physiological fluids, liver microsomal stability, hepatocyte stability, plasma protein binding, microsomal partitioning, CYP450 inhibition, and permeability. The center’s consumable and personnel costs are covered by funding sources from grants and contracts. The intellectual property policy is university driven and supported by Temple University’s Office of Technology Transfer.

Website: https://moulder.temple.edu/

Email: aboumag@temple.edu

The **Chemical Biology & Therapeutics** group (CBT) is located at the medical faculty of Lund University, Sweden, and the **Chemical Biology Consortium** (CBCS) is located at Karolinska Institutet, in Stockholm, Sweden.^9^ The CBT was established in 2013 with financial support from Lund University to support its researchers. The facility’s personnel are funded by MultiPark (a strategic research area at Lund University with translational focus on Parkinson’s and Alzheimer’s disease) and projects are managed as academic collaborations (sharing costs and intellectual property [IP]). The facility currently has Bravo BenchCel liquid handling systems with a PlateLoc micropate sealer from Agilent (Santa Clara, CA) and MultiDrop Combi from ThermoFisher (Waltham, MA). The facility uses various technologies for screen readouts (fluorescence-activated cell sorting [FACS], high-content screening [HCS], plate readers, etc.) in collaborations with other infrastructures at the faculty. The large number of assay modalities allows the CBT to be very flexible in the types of assays it can process. The CBCS is a national infrastructure for chemical biology within the Science for Life Laboratory. Its operations began in 2010, first funded by the Swedish Research Council and currently by the Science for Life Laboratory and the host universities (Karolinska Institutet and Umeå University). At CBCS, 12 scientists with a background in the pharmaceutical industry (medicinal chemists, biochemists, cell biologists) are employed. CBCS offers instrumentation and experience in performing most types of high-throughput screens (biochemical, cell-based reporter system, phenotypic cell-based as well as image-based high-content screens). Through CBCS, academic researchers have access to ~200,000 small molecules, including a set of known drugs and annotated compounds. CBCS performs chemical screens and has suitable liquid handling equipment and technologies for most assay readouts. Access to CBCS is open on equal terms to all Swedish researchers (academic, healthcare, and industry). Swedish researchers are prioritized over international users. The cost model has the Swedish academic researchers pay for consumables and compound access. The researchers only partially pay for expertise during assay development, screening, and follow-up work. The CBCS general user agreement does specify any IP rights; however, CBCS does not intend to claim rights and has, upon request of users, transferred IP rights.

Websites:

CBT: http://portal.research.lu.se/portal/en/organisations-researchgroups/chemical-biology-and-therapeutics(a49506d7-e018-4ccb-b701-f3f1ff02fcfb).html

Email: Roger.Olsson@med.lu.se

CBCS: http://www.cbcs.se

Email: Anna-Lena.Gustavsson@ki.se

The **Centre for Integrative Biology High-Throughput Screening and Validation** (CIBIO-HTS) facility has been operating since 2011 within the University of Trento, Italy.^14^ CIBIO infrastructure includes nine state-of-the-art core facilities, all independent from the university’s research groups and run by qualified personnel. The CIBIO-HTS facility supports the development of screening programs and offers an interactive and open environment to researchers willing to apply chemical and functional biology or image-based cytometry to their research. The facility is staffed with four people with many years of experience. Close collaboration with 40 internal research groups generated core competencies in oncology and neurobiology. Collaboration with other CIBIO core facilities allows the facility to expand its expertise to stem cells, zebrafish, and *Drosophila*, as well as to protein production and biophysical characterization of molecules and interactions. The facility can handle a wide variety of experimental setups, from low-throughput drug testing to higher-throughput screenings, using automated liquid handlers (Tecan Evo200, Tecan Group, Männedorf, Switzerland), BioTek EL406 (BioTek, Winooski, VT), multimode plate readers (luminescence, fluorescence, AlphaScreen, DMR Epic Label-Free), imagers (PE Operetta and Ensight, PerkinElmer, Waltham, MA), and other multi-well-based detection systems (Acea XCelligence, SeaHorse, Agilent, Santa Clara, CA). Long and broad experience in high-content screening allows flexibility in the choice of the model to be screened, from 2D and 3D cultures (i.e., spheroids and organoids) to whole organisms, for both target-based investigations and multiparameter phenotypic profiling. The commercial libraries available include drugs or drug-like molecules selected for their known pharmacological and biological effect and for their chemical diversity. The CIBIO-HTS facility has acquired skills in a broad range of screening projects, both biochemical and cell based, with the focus on RNA biology and posttranscriptional processing. The reporter-based cellular assays (luciferase or fluorescent proteins) are applied to challenge the regulatory processes of mRNA, such as splicing, export, localization, turnover, and translation efficiency. Novel biochemical assays employing AlphaScreen and DMR Epic Label-Free technologies have been developed to study RNA–protein interactions. Finally, the facility teams up with other internal infrastructures to provide validation strategies and techniques such as transcriptomics and proteomics and supports quantitative and droplet digital PCR and polysomal fractionation analysis. The use of an electronic booking system to track the usage of assets (instruments, staff, services, consumables) and the application of access rules, as well as a detailed analysis of the costs associated with the facilities, allows opening access to internal and external academic users, as well as private companies. CIBIO-HTS also provides assistance for grant applications by drafting experimental designs and providing letters of support. In case of publications or new patent applications, co-authorship or joint inventorship may be considered appropriate only if the staff have provided a significant intellectual contribution to the assay development or the hit validation.

Website: https://www.cibio.unitn.it/48/high-throughput-screening-hts-and-validation

Email: hts.cibio@unitn.it

**LifeArc**, formerly known as MRC Technology, is an independent medical research charity.^15^ Founded in 2000, MRC Technology brought together the different groups involved in the translation of UK Medical Research Council-funded science. In 2001, the assay development group was formed to work with academic scientists to generate reagents and assays to allow plate-based screening of novel targets. It was quickly realized that to interrogate targets further, quality chemical probes were required, and that screening alone was unlikely to deliver these. In 2005, a chemistry group was added with a focus on medicinal chemistry, developing chemical tools needed for target validation and developing early-stage chemical hits into chemical leads as a basis for collaborations or licensing deals with pharma and biotech partners. This gave rise to LifeArc’s Centre for Therapeutics Discovery (CTD). LifeArc now works independently of the MRC and is funded by successful outputs of licensing (e.g., royalties from the humanization of antibodies such as Tysabri, RoActemera, Entyvio, and Keytruda). Since 2016, CTD has been based in Stevenage (UK) and collaborates with academic groups around the world. There are more than 90 scientists working in early drug discovery, including target validation, pharmacology, assay development, screening, antibody engineering, medicinal chemistry, computational chemistry, and early ADME. Screening formats include cellular (target-specific and phenotypic), biochemical, and increasingly, biophysical screening. LifeArc has an in-house compound collection including a diversity (125,000 compounds) and an indexing set, as well as several focused sets (kinase, ion channel, protein–protein interaction (PPI), fragments, natural products, annotated). LifeArc’s model is to form active collaborations with academic groups in the early drug discovery space. Early interactions are key to shaping a project in terms of validation experiments, advice on assay development, and the sharing of compound libraries. When a project moves into the LifeArc portfolio, a collaboration agreement with the host institution is put in place determining governance and intellectual property ownership and a project team is formed consisting of LifeArc and originating lab personnel. LifeArc covers all the costs associated with LifeArc activities and can work with an academic group to secure funding for aspects ongoing in the originating lab.

Website: https://www.lifearc.org/working-with-us/drug-discovery/

Email: info@lifearc.org

The **Fraunhofer-Institute IME** in Hamburg, Germany, began its operations in 2007 (from 2007 to 2014, the organization operated as European ScreeningPort GmbH) and currently employs 25 employees.^12^ The IME operates two fully automated screening systems. The first system is a PerkinElmer Cell::Explorer system (PerkinElmer, Waltham, MA), which has a modular design and contains integrated liquid handling, compound reformatting, incubators, a high-throughput microscope, and a high-throughput multimode plate reader for the commonly used screening assays. The second system is the Tecan Fluent 760 (Tecan Group, Männedorf, Switzerland), which is a compact and fast system for automated screening. The screening technologies and readouts available include AlphaScreen, time-resolved fluorescence resonance energy transfer (TR-FRET), fluorescence intensity, confocal high-content imaging, absorbance, luminescence, reporter gene, cellular biosensors (e.g., Fucci cell cycle, caspase induction, cAMP), immunocytochemistry, cell migration and translocation, ion sensing (e.g., Fluxor, Fluo4, Fura2), enzyme-linked immunosorbent assay (ELISA), proximity ligation analysis, cell-free electrophysiology, label-free dynamic mass redistribution, surface plasmon resonance, and fluorescence polarization. Most assays are screened against compound libraries, and we have access to some 500,000 small molecules and 10,000 natural products. The target classes the IME has experience with include kinases, G-protein-coupled receptors (GPCRs), ion channels, histone demethylases (HDACs), phosphodiesterases (PDEs), synthases, transferases, deubiquitinylases, protein–protein interactions, transporters, efflux pumps, proteases, noncoding RNAs, transcription factors, nuclear hormone receptors, mitochondrial membrane channels, DNA binding proteins, and toxicity readouts (CYP450s, micronucleus gene toxicity assays, *h*ERG, and mitochondrial toxicity). The IME operates under an open-access model with internal and external users under a research collaboration contract using a full-cost recovery model. Where appropriate, intellectual property is shared between users and the platform depending on the respective contributions and contract conditions.

Website: https://www.ime.fraunhofer.de/en/Research_Divisions/business_fields_TM/screeningport.html

Email: philip.gribbon@ime.fraunhofer.de

Within the **Bio-Analytical Chemistry** division at the Vrije Universiteit Amsterdam in the Netherlands, Dr. Jeroen Kool works as an analytical chemist with research interests in high-resolution screening of biologically active mixtures.^13^ His research achievements allow full compatibility of analytical separations with biological assays (including cellular) and parallel mass spectrometry (MS) detection for investigation of bioactive mixtures (i.e., natural product extracts, lead compound metabolic mixtures, venoms, environmental mixtures, and food products) using miniaturized setups and nanospotting technologies. These techniques combining chromatography, MS, and bioassays in one analytical platform are now known as nanofractionation analytics. In recent years, hyphenated analytics for both liquid chromatography (LC) and gas chromatography (GC) separations to bioassays for the identification of biologically active toxicants in natural extracts, food, and the environment have been developed. For GC fractionations, Kool developed and patented an automated system for high-resolution fractionation of complete gas chromatograms with parallel chemical detection, such as flame ionization detection (FID) and MS. Kool also developed analytical methodologies for bioactivity profiling of metabolic mixtures from drugs and lead compounds targeting G-protein-coupled receptors (GPCRs), nuclear receptors, protein kinases, and ion channels. Currently, Kool is working on postcolumn microfluidics and nanospotting analytics for the analysis of bioactive mixtures only available in low amounts. Insect and animal venoms are examples that contain many different, highly potent, and sometimes very selective peptide ligands for a large variety of medicinal targets. Some of the analytics and other techniques in the Bio-Analytical Chemistry division include nano-LC-MS systems, nanofractionation analytics (terminology we introduced to analytical science), high-resolution screening analytics, GC fractionation equipment (patented by us and now commercially available via our partner Da Vinci), and LC fractionation bioassay analytics (now commercially available via SPARK-HOLLAND and Vrije Universiteit). Typically, bioassays use existing formats and implement them in the laboratory’s analytics, or they are developed from scratch if they are not available. The bioassays range from enzymatic to ion channels, nuclear receptors, GPCRs, cellular assays, and other assay formats. Kool’s laboratory has ample expertise in capillary electrophoresis (CE)-MS analytics and started working with matrix-assisted laser desorption/ionization (MALDI)-MS imaging projects recently. One of the research efforts is directed at trapped ion mobility spectrometry (TIMS) looking at metabolites of drugs. Fifteen MS systems (high and low resolution), 10 CEs, 3 nano-LCs, 3 ultra-pressure liquid chromatography (UPLC) systems, 40+ high-pressure liquid chromatography (HPLC) systems, several GCs, an HTS lab, a cell culturing lab, and access to a radio nucleotide lab are available. Dr. Kool’s core expertise is in coupling separation techniques with MS and bioassays to identify the bioactive components in complex mixtures. The laboratory is open to discuss possibilities for any form of access/cost model, ranging from full collaborations to full fee-for-service models.

Website: https://research.vu.nl/en/persons/j-kool

Email: j.kool@vu.nl

After initially locating at the Florida Atlantic University Honors College in Jupiter, Florida, in 2005, the **Scripps Research Molecular Screening Center** (SRMSC) found its permanent home in a brand-new facility built in 2008.^11^ It currently comprises more than $22 million of specialized automation for early drug discovery and screening. The Translation Research Institute (TRI), under the direction of Patrick Griffin, was founded at approximately the same time. Together with researchers in the Rosen Lab in La Jolla, California, at Scripps Research, about 15 employees represent one of the most competitive academic industrial screening centers worldwide. The SRMSC houses two fully automated Kalypsys/GNF (Genomics Novartis Foundation, San Diego, CA) platforms, one a screening platform and the other a cherry-picking platform. Additionally, matching technologies are appended within the SRMSC to allow scientists and engineers to develop assays and prepare them for automated screening. The SRMSC is a small-molecule screening center and archives more than 1 million compounds, including a proprietary collection of >665,000 molecules derived from commercial sources from around the world. As part of this collection, medicinal chemistry has included ~40,000 unique compounds that are not found elsewhere. These collections are screened against a myriad of disease targets, including cell-based and biochemical assays. Scripps has proven competence in a broad range of detection formats, including high-content analysis, fluorescence, bioluminescence resonance energy transfer (BRET), time-resolved fluorescence resonance energy transfer (TR-FRET), fluorescence polarization (FP), luminescence, absorbance, AlphaScreen, and Ca^++^ signaling, to name a few. These technologies are applied to both NIH-derived academic collaborations and small- and large-scale biotech and pharma initiatives. The SRMSC and TSRI are proud to have been able to provide full-cost recovery since their inception and are recognized for generating intellectual property impacting the benefit of mankind (see Ozanimod).

Website: https://hts.florida.scripps.edu/

Email: SRIMSC@scripps.edu

**EU-OPENSCREEN**, the European research infrastructure for chemical biology, was founded in April 2018 with the support of 21 screening and medicinal chemistry partner sites from eight European member and observer states and the European Commission.^16^ EU-OPENSCREEN is headquartered in Berlin, and its main goal is to facilitate the development of new molecular research tools and drug candidates—thus ensuring European competitiveness in the fields of chemical biology and early drug discovery. The EU-OPENSCREEN compound collection of 140,000 molecules can be accessed by collaborators from academia and industry and represents one key physical asset of the network. Its commercial part comprising 100,000 small molecules and fragments from both large and smaller, more specialized vendors is carefully designed and assembled by five EU-OPENSCREEN partner institutions to ensure high diversity and distinctiveness of the library (e.g., sp^3^ richness and compounds with potential for polypharmacology). In addition, the academic part comprising up to 40,000 molecules, crowd-sourced from European chemistry laboratories over time, will further add unique chemistry and new molecule classes such as natural product-like compounds to the collection. Hereto, expertise regarding isolation and chemical characterization of marine and microbial natural products is well represented within the network. All compounds of the EU-OPENSCREEN compound collection are bioprofiled for physicochemical properties such as solubility, potential for interference with certain assay readouts, and cellular toxicity, at designated partner institutions. Bioprofiled data and structural information are stored in the open-access European Chemical Biology Database (ECBD). Incoming projects are assigned to the most suitable of the 15 EU-OPENSCREEN screening partners based on individual project needs. Due to its distributed character, EU-OPENSCREEN as a collective covers a plethora of specialized project needs alongside standard phenotypic and target-based high-throughput and high-content screening approaches. These include fragment-based screening by nuclear magnetic resonance (NMR) and surface plasmon resonance (SPR), high-throughput mass spectroscopy screening, high-throughput flow cytometry methodologies for cell-based screens, screening under BSL2/3 conditions, protein–protein interaction screens, multiplexed biochemical and imaging assays, combinatorial screening of compounds, target-based biophysical screening, in vitro and in vivo ADME profiling, and virtual screening for hit analogues. Importantly, innovative cellular systems such as human organoids, induced pluripotent stem cells, or patient-derived cells can be readily supported. After the initial screen, collaborators can rely on EU-OPENSCREEN’s six medicinal chemistry partners for chemical optimization of hit compounds. Expertise within the network includes the design, synthesis, and chemical optimization of compounds and fragments with a high degree of three-dimensionality and stereochemical diversity, as well as structure–activity relationship analysis during chemical optimization. In conclusion, due to EU-OPENSCREEN’s interdisciplinary, collaborative working model, collaborators can run their entire project within the network and benefit from the collective expertise of the 21 partner sites. Biological assay data will be made publicly available in the ECBD after a grace period of up to 3 years, which is meant to ensure publication and/or patenting of experimental results. EU-OPENSCREEN is a not-for-profit organization, and costs are calculated to only cover the replenishment of used compounds and consumables at partner institutions. Collaborators from industry will be charged a 20% surcharge. A more detailed description of the EU-OPENSCREEN network and its participating partner institutes can be found online.

Website: https://www.eu-openscreen.eu/

Email: office@eu-openscreen.eu

## Conclusions

While this special issue offers just a glimpse into the work going on in the academic and not-for-profit drug screening world, it illustrates a rich landscape that offers a great diversity and a range of expertise. For more information and to connect more closely with this exciting community, readers of this special issue are encouraged to join the SLAS Americas Academic Drug Discovery Special Interest Group online at https://www.linkedin.com/groups/4083717 and/or the SLAS European Academic Drug Discovery Special Interest Group online at https://www.linkedin.com/groups/8417733. Members of these special interest groups also host meetings during the annual SLAS International Conference and the annual SLAS Europe Conference to discuss topics of interest and network with other professionals in this field.
